# Physicochemical and Microbiological Characteristics of Stem Bark Exudate Gum of *Cordia millenii* Tree in Conventional Release Tablets

**DOI:** 10.1155/2023/9118067

**Published:** 2023-05-04

**Authors:** Samuel Lugrie Kipo, Kwabena Ofori-Kwakye, Noble Kuntworbe, Raphael Johnson, Mariam El Boakye-Gyasi, Yaa Asantewaa Osei, Frederick Akuffo Owusu

**Affiliations:** Department of Pharmaceutics, Faculty of Pharmacy and Pharmaceutical Sciences, College of Health Sciences, Kwame Nkrumah University of Science and Technology, Kumasi, Ghana

## Abstract

The development of a raw material into an acceptable pharmaceutical excipient involves evaluation of the physicochemical and formulation properties of the potential raw material. Results from these evaluations may serve as a guide to subsequent use of the substance. The objective of the study was to evaluate the physicochemical and microbiological properties of the stem bark gum of *Cordia millenii* tree in conventional release paracetamol tablets. From the physicochemical evaluations, the gum was slightly acidic and soluble in all the aqueous-based solvents, except 0.1 N HCl in which it was sparingly soluble. All the absorptive properties of the gum indicated tablet disintegrating potential for tablet formulation. The total ash of the gum was higher than that of the international standard gum arabic. Micromeritic properties of the gum indicated the need for a flow aid to improve its flowability. There were no harmful microorganisms detected in the gum. Aerobic organisms and moulds and yeast were detected within permissible limits. Tablets formulated using six different concentrations of gum dispersions as a binder were generally soft and failed the USP *T*_80_ standard of dissolution, indicating poor binding and drug releasing properties. Quality control properties of three different batches of tablets containing varying concentrations of the dry gum as a disintegrating agent were comparable to tablets containing equal concentrations of corn starch. The in vitro drug releases were similar at all-time points of drug evaluation. The gum can therefore be considered as a good disintegrant in the formulation of conventional release tablets.

## 1. Introduction

Exudate gum is one of the two types of plant-based gums obtained from trees and shrubs [1], usually by breakdown of cell walls of certain plants, following injury caused by insect borers, human activities (tapping), and diseases and/or under adverse growth conditions such as drought, bushfires, or soil conditions [2–8]. However, the process of gummosis is suggested as a mode of protective mechanism by sealing off the site of injury against microbial infection and/or excessive loss of water after injury and, therefore, not normal physiological product of metabolism [9–11]. Plant gums are hydrosoluble or hydrogel complex nonstarch polysaccharide polymers which are very versatile in application. Most of the functional properties of plant gums depend on the viscosity and swelling properties [6, 12]. Gums are ideal excipients of diverse uses in a pharmaceutical formulation of conventional and novel drug delivery systems [7, 13–15]. In tablet formulation, gums have been used as binders, solubility enhancers, matrix formers or drug release modifiers, film coating formers, and disintegrants [2, 16, 17]. Plant-derived polymers comply with many requirements of pharmaceutical excipients. Gums are usually described as being biodegradable and biocompatible with no or low toxicity [7, 13, 18]. Because gums are natural products, they are obtained from renewable source and extraction and/or purification processes pose no risk to the environment. Gums are also characterized by low or no side effects and, therefore, have better patient tolerance and acceptability. Natural gums are easily available worldwide and economical to use, hence contributing to improving the national economy by providing inexpensive formulations to people [17, 19, 20].

As the use of excipients in drug delivery, especially in the preparation of novel products (modified drug release dosage forms) increases, the search for suitable and cost-effective and newer natural excipient alternatives becomes necessary [21–27].

*Cordia millenii*, commonly called drum tree or African Cordia, from the family Boraginaceae, is a well-distributed tropical African forest tree that can grow up to 20 m, and in some regions (Uganda), it is 40 m high and has a bole diameter of about 1 m or more [28, 29]. It is called omo in Yoruba (Nigeria), omah in Benin, because of its use in making talking drums [30, 31]. Major uses of *Cordia millenii* tree are in the area of timber industry and traditional herbal medicine [30, 32, 33]. In African phytomedicine (where it is an indigenous plant), various parts are used to treat or manage fevers, cough, stomachache, toothache, inflammatory-related disorders, HIV, diarrhea, dysentery, ringworm, and worm infestation [30, 33–36].

Phytochemicals isolated from different parts of *Cordia millenii* plant include glycosides (triterpenoids), resins, lipids, sugars, alkaloids, essential oils, tannins, flavonoids (phenolic compounds), steroids, saponins, terpenoid benzoquinones called cordiachromes A–F, and the latest being cordidepsine [29, 34, 36, 37]. When the stem is slashed, viscous liquid oozes out freely after 7–10 days and continues for several weeks. When this viscous liquid is allowed to dry on the tree, it forms a broad mass with finger-like ribbons.

The objective of this study is to evaluate the physicochemical and conventional tablet formulation properties of this copious oozing gum from the stem bark of *Cordia millenii* tree as a binder or disintegrant. Studies carried out on the gum have shown that the gum does not pose any toxicity to humans and is a potential candidate for use as a pharmaceutical excipient in drug formulations [38].

Depending on the ingredients of a solid dosage form such as a tablet, disintegration may proceed to granules and granules deaggregate into primary drug particles. The process of tablet dissolution can occur from any or all of these at different rates. Depending on the efficiency of a binding and/or disintegrating agent, dissolution may occur at a slow rate from an intact tablet, at a moderate rate from granules, and/or at a relatively rapid rate from primary drug particles [39]. Therefore, dissolution rate and parameters will be used in this study to evaluate the efficiency of the gum as a binder or disintegrant.

## 2. Materials and Methods

### 2.1. Materials

The partially dried stem bark gum of *Cordia millenii* was collected from Miaso Kwahu in the eastern region of Ghana. Leaves and branch parts of this plant were also harvested for identification and confirmation at the Department of Herbal Medicine, College of Health Sciences, KNUST, Ghana. Lactose and corn starch were gifts from Aspee Pharmaceuticals (Kumasi, Ghana); polyvinylpyrrolidone (PVP) and magnesium stearate were gifts from Poku Pharmaindustry (Kumasi, Ghana), the model drug; paracetamol (acetaminophen) powder and all the required laboratory grade reagents were obtained from the chemical stores of Departments of Pharmaceutics and Pharmaceutical Chemistry, KNUST, Kumasi. All solutions were prepared with distilled water and were freshly used.

### 2.2. Methods

#### 2.2.1. Purification of Gum

The partially dried gum collected was visually sorted and all nongum materials were removed. The crude gum was dried in a hot air oven at 50°C till it became sufficiently brittle. The gum was further cleaned by manual scrapping of pieces of bark and other extraneous materials that were not detected before drying. This was comminuted through sieve #18 and purified by the modified method of Ofori-Kwakye et al. [40].

A weight of 250 g was transferred into 6x its weight (6 × 250 ml) of distilled water for 72 h with occasional stirring for hydration and dissolution. This mixture was strained with calico linen, and the filtrate was refiltered to ensure that all debris were removed and the volume was adjusted to 1000 ml. The gum was precipitated with 1.6 volumes of filtrate (1.6 × 1000 ml) of 96% ethanol. The precipitate was completely dissolved in sufficient (about 300 ml) distilled water, precipitated again with sufficient (375 ml) 96% ethanol, and washed in about 100 ml of diethyl ether and left to stand on the bench for 2 h. It was then dried in a hot air oven at 50°C for 24 h and weighed. The percent yield was calculated using the following formula:

(1) $$\begin{array}{c} Yield\;\%=\frac{weight\;of\;purified\;gum}{weight\;of\;crude\;gum}x\;100\text{.} \end{array}$$

The gum was powdered using a mortar and pestle and sieved through sieve #80, packed in an airtight container, and labelled as purified *Cordia millenii* gum (pCmG).

This was confirmed by the ruthenium red test of Choudhary and Pawar [41].

#### 2.2.2. Phytochemical Screening of pCmG

The pCmG was screened qualitatively for primary and secondary metabolites.

The primary metabolites screened for were carbohydrates by Molisch's reagent test [41], starch by the iodine test [42], and reducing sugars by Benedict's test solution [43]. The secondary metabolites tested for were alkaloids with Dragendorff's reagent [37], glycosides by the bromine water test [44] and Keller Killiani test [45], saponins by the froth test [46], steroids by the ethanol and conc. H_2_SO_4_ test [47], terpenoids by Salkowski's test [45], and coumarins by the fluorescence test [48].

Other secondary metabolites tested for were tannins by dilute lead acetate and ferric chloride solutions [44, 47] and protein and free amino acids using biuret and ninhydrin tests, respectively [44, 49]. Flavonoids were tested for by alkaline reagent and ferric chloride solution [44].

#### 2.2.3. Physicochemical Properties of pCmG

*(1) Weight Loss on Drying (Moisture Content) and Insoluble Matter Content*. Triplicate determinations by BP [50] procedures for acacia gum were used.

*(2) Solubility Profile and pH*. This was evaluated in distilled water, 0.1 N HCl, phosphate buffers of pH 6.8 and 7.4, chloroform, ethanol (96%), and acetone, at room temperature (25°C). Distilled water evaluation was repeated at 80°C. Three determinations were done for each solvent and expressed in each solvent as the approximate weight (g) of solvent necessary to dissolve 1 g of pCmG at 25°C [50].

The pH of pCmG was determined using a freshly calibrated Milwaukee 101 pH meter.

*(3) Hydration, Swelling, and Water Retention Capacities*. The method adopted by Akpabio et al. [51] was used with some modifications. Three 1 g portions of the gum were placed in previously weighed (*w*_0_) different 15 ml graduated centrifuge tubes, and each was tapped (200 times) till consistent volume (*v*_1_) was obtained. Each centrifuge tube containing gum powder was weighed (*w*_1_), and 10 ml of distilled water was added into each tube and shaken vigorously for 5 min to obtain uniform dispersion. The tubes and contents were left for 10 min and centrifuged at 4000 rpm for 10 min. The supernatant liquid on each sediment was carefully decanted into a 10 ml measuring cylinder and the volume was noted (*v*_*f*_). The volume of the sediments was recorded as *v*_2_, and tubes and content were weighed, *w*_2._

All the capacities were expressed in percentages as hydration capacity, HC = [(*w*_2_ −  *w*_1_)/(*w*_1_ − *w*_0_)] × 100, swelling index (capacity), (SI) = [(*v*_2_ − *v*_1_)/*v*_1_] × 100, and water retention capacity (WRC) = [(10 − *v*_*f*_)/10] × 100.

This procedure was repeated for 0.1 NHCl, pH 6.8, and 7.4 phosphate buffer solutions.

*(4) Moisture Sorption/Desorption Capacity*. The method of Eddy et al. [52] was used with some modifications. Three plastic tops were dried at 105°C and cooled in a desiccator, and each was weighed. About 6 g of pCmG powder was also dried at 50°C for 1 h and 1 g was weighed into each plastic top and reweighed. Plastic tops and contents were placed in water in a desiccator and each were weighed daily (24 h) for 7 days (when almost consistent weight was observed). The tops and contents were transferred into another desiccator containing activated desiccant (dry silica gel) and weighed daily (as above) for 6 days. The percentage sorption/desorption for each day was obtained as the quotient of mean weight difference per dry gum weight multiplied by 100. This was plotted against the number of days.

*(5) Inorganic Content of pCmG Powder*. This was evaluated by determining the total ash, water- insoluble ash, and acid-insoluble ash of the pCmg powder sample.

  Total ash value:
  Triplicate determinations of total ash value were done using the BP [50] method for determining sulfated ash with some modification. Three ash crucibles were heated to redness in a hot air oven for 30 min and allowed to cool in a desiccator. The crucibles were quickly weighed (A) and three portions of 2 g of gum were placed in each crucible and spread evenly. Each crucible and content were dried at 105°C for 1 h and transferred into a furnace for 4 h at 550°C. The furnace was allowed to cool to about 180°C and maintained for 20 min. The crucibles and content were carefully transferred into a desiccator, cooled, and reweighed (C). The % total ash value of the gum sample in each crucible was calculated as [(C − A)/2 g (weight of gum)] × 100, and the mean was determined.
  Water-insoluble ash:
  The total ash in each was carefully washed with 25 ml of hot distilled water into, respectively, labelled beakers and each was boiled gently for 5 min. The mixtures were each filtered through an ashless filter paper, and each beaker was washed thoroughly with hot distilled water on their respective ashless filter papers. Each ashless filter paper was allowed to drain completely and carefully transferred to respective crucibles, dried at 105°C, and ignited at 550°C in the furnace for 4 h. The furnace was allowed to cool to about 180°C and maintained for 20 min. The crucibles and contents were carefully transferred into a desiccator, cooled, and reweighed (D).
  The % water-insoluble ash of the gum sample in each crucible was calculated as [(D − A)/2 g (weight of gum)] × 100, and the mean of the three crucibles was calculated as water-insoluble ash of the gum [53, 54].
  Acid-insoluble ash:
  For the acid-insoluble ash, the BP [50] method was used with some modifications from other researchers [53, 55]. Procedure for determining total ash was repeated and proceeded by carefully washing the ash in each crucible with a mixture of 15 ml distilled water and 10 ml HCl (3 : 2) into labelled beakers. Each beaker was covered with watch glass and boiled gently for 10 min and allowed to cool. The bottom of each watch glass was rinsed with 5 ml hot distilled water into each beaker. Each acid solution was filtered through an ash-less filter paper and the process continued as was done for water-insoluble ash.

Ethanol-insoluble ash was also determined as done for water-insoluble ash, except that the boiling process of 96% ethanol washings was done using a reflux condenser.

#### 2.2.4. Microbial Properties of pCmG

Microbial viable count analysis using the plate count and agar well diffusion methods described by Das et al. [56] was used.

#### 2.2.5. Micromeritic Parameters of pCmG

*(1) Bulk Densities, Hausner Ratio, and % Compressibility Measurements*. Thirty grams (30 g) of pCmG powder (sieve #80) were carefully transferred into a 100 ml measuring cylinder tilted at about 60°. The cylinder was straightened and the granules cautiously levelled in the cylinder without disturbing the packing of the entire powder. The volume of the granules was recorded as the initial volume, *V*_o_.

The initial (fluff or poured) bulk density, *D*_o_, was then determined as the ratio of powder mass (M) to the initial volume (*V*_o_).

That is, the initial/fluff/poured bulk density, *D*_o_ = *M*/*V*_o_.

The cylinder and content were raised through a distance of about 5 cm and allowed to fall on a hard pad (200 times) until constant volume *V*_*f*_ was obtained.

The final (equilibrium/tapped/consolidated) density, *D*_*f*_, was determined as *M*/*V*_*f*_.

Hausner ratio was evaluated as the quotient of tapped density, *D*_*f*_, and initial bulk density, *D*_o_ (*D*_*f*_/*D*_o_), and % compressibility (Carr's index) was also calculated as {(*D*_*f*_ − *D*_o_)/*D*_*f*_)} × 100.

*(2) Angle of Repose*. The fixed height cone method was employed. A funnel with the stem cut flat was clamped about 10 cm above a graph paper spread on a flat surface. Fifty grams (50 g) of the gum powder were poured through the funnel to form a cone on the graph paper. The funnel was carefully adjusted for the tip of the stem to touch the apex of the cone. The funnel was firmly clamped and the gum powder was collected. The procedure was repeated such that the apex of the cone just touched the tip of the stem of the funnel. The base of the cone on the graph paper was marked severally and the diameter, d, measured after the powder was collected. The height, h, of the cone was measured as the tip of the stem of the funnel to the graph paper. The angle of repose was calculated as the tangent of the angle ө = 2 h/d. The entire procedure was repeated three times for each gum.

#### 2.2.6. Binding and Disintegrating Potentials of pCmG in Conventional Release Tablets

(1) *Gum-Drug (Paracetamol) Compatibility Testing*. The diamond crystal of the Bruker IR-ATR (infrared attenuated total reflectance) spectrophotometer (Model Bruker Alpha Platinum ATR) was cleaned with isopropanol and a background scan was taken. A small quantity of pCmG powder was placed directly on the diamond crystal, and the pressure gauge was applied until maximum contact was obtained. This gum sample was scanned through a midwave number of 400–4000 cm^−1^. This process was repeated for the paracetamol powder alone and the mortar blend of paracetamol-pCmG powder in a ratio of 1 : 1.

Percent quantity of paracetamol in the mixture was determined using the BP [50] procedure of assaying paracetamol tablet. The rest of the powder mixture was transferred into a sample bottle and stored at 40 ± 2°C and 75% relative humidity in a stability chamber for 30 days [57]. Determination of percent drug content was repeated, and the IR spectrum was generated again. The spectra were compared for any possible interaction(s) after mixing and/or storage.

*(2) Use of pCmG as Tablet Binder*

1. Formulation design and granulation:
2. Six different tablet formulation batches each containing equivalent pCmG concentrations of 0.5, 1.0, 2.0, 3.0, 4.0, and 5.0% w/w were prepared and labelled as Cm0.5, Cm1.0, Cm2.0, Cm3.0, Cm4.0, and Cm5.0, respectively (Table 1). Dispersions of pCmG were used in each batch.
3. Six different batches of granules were prepared using the wet granulation method with #12/20 sieves. Flow properties of granules comprising bulk densities, Hausner ratio, % compressibility indices, and angle of repose were all evaluated using procedures used for pCmG powder. Each batch of granules was mixed thoroughly with 0.35% w/w of magnesium stearate and manually compressed with Hanseaten *E*_1_ (Wilhelm Fette) single punch tableting machine within the compaction force of approximately 75–80 N (7.5–8.0 kgf).
4. Quality assessment of the compressed tablets:
5. Drug content, uniformity of weight, resistance to crushing, and disintegration tests were determined by procedures outlined in BP [50] for compressed tablets. The dissolution profile was determined using BP [50] and USP 30-NF 25 (2007) apparatus II, the paddle method. The dissolution medium was 900 ml of pH 5.8 phosphate buffer and a paddle speed of 50 rpm. The dissolution of each batch of tablets was done by the USP 30-NF 25 (2007) procedure under sink conditions. Ten milliliters of the dissolution medium and drug mixture were withdrawn, filtered immediately, and replaced with fresh dissolution medium at 2.5, 5, 10, 15, 30, 45, and 60 min. The filtrates were diluted with phosphate buffer pH 5.8 (dissolution medium) and absorbance was determined. Before this determination, the UV spectrophotometer was calibrated with a series of standard solutions of freshly prepared paracetamol with the dissolution medium. A regression line (from the calibration) was used to calculate the actual amounts of paracetamol at each time point as the drug was released and plotted against time.

The USP 30-NF 25 (2007) tolerance of 80% of labelled paracetamol content (500 mg) to dissolve within 30 min [*T*_80_(min)] and model independent parameter and dissolution efficiency (DE) were estimated and compared. DE was estimated using the formula {(0∫^t^*Y*.d*t*)/Y%.(*t*_2_ − *t*_1_)} × 100.

(0∫^*t*^*Y*.d*t*) = area under the dissolution curve (AUC).

*Y* = 100% dissolution at *t*_2_.

*t*_2_ = time for all active ingredients to dissolve.

*t*_1_ = time at which the first sample (dissolution medium and drug mixture) was withdrawn [58]. The dissolution efficiencies were calculated with a constant time interval and compared.

*(3) Use of pCmG Powder as Tablet Disintegrant*.  Table 2 contains master formula of 2 groups of 3 batches of paracetamol tablets. Each group contained 3 batches of pCmG and corn starch (reference) powders as disintegrating agents. These batches were labelled as Cm6, Cm8, and Cm10 and ST6, ST8, and ST10, representing pCmG (Cm) and corn starch (ST) groups, respectively. The numbers 6, 8, and 10 attached to the tablet batches represented the %w/w of pCmG and corn starch per tablet.

An appropriate volume of 20% w/v PVP was used as a binder solution to granulate each formulation such that the concentration of PVP was 2.5% w/w per tablet. The granules were assessed, lubricated with 0.35% w/w of magnesium stearate, and compressed into tablets. The physical properties and dissolution parameters of the compressed tablets were determined. The dissolution parameters were compared statistically by the model independent mathematical approach; the fit factors connoted by *f*1 and *f*2 represent difference and similarity factors, respectively.

(2) $$\begin{array}{c} The\;difference\;factor\;computed\;as\;f1=\left\{\frac{\sum \left|Rt-Tt\right|}{\sum Rt}\right\}x\;100\text{,}\\ similarity\;factor,f2=50\;\log\;\left\{{\left[1+\left(1/n\right)\underset{\left(t=1\right)}{\sum }{*\;n\;\left(R_{t}-T_{\left(t\right)}\right)}^{2}\right]}^{\left(-0.5\right)}\;*100\right\}\text{,} \end{array}$$

where *n* = number of sampling time points *t*, *t* = 1 to *n*, R_t_ = cumulative percentage dissolved at time t for the reference, and Tt = cumulative percentage dissolved at time t for the test [59].

## 3. Results and Discussion

The mean percentage yield of pCnG was 38.5%, which was relatively low; however, its pharmaceutical acceptability as an excipient may depend on the physicochemical and formulation characteristics. All the organoleptic properties of the gum were good for use as a pharmaceutical excipient, except the colour which may likely be a slight disadvantage to its use (especially in higher concentrations) in colourless formulations (Table 3).

The positive ruthenium red test confirmed pCmG as a plant gum [60, 61]. Apart from the primary metabolites, carbohydrates and reducing sugars, starch and all the secondary metabolites tested for were absent. This is a confirmation that gums are not products of metabolism [62] and also attests to the efficiency of the purification procedure adopted. The absence of these secondary metabolites in pCmG is an advantage to its use as a pharmaceutical excipient.

The loss on drying (LOD) and insoluble matter of pCmG were lower than acacia (BP) [50] with values of 10–15 and 0.5%, respectively. The relatively high insoluble matter value may suggest the presence of high levels of inorganic matter in the gum, and this was confirmed by the total ash value which was higher than that of the international standard gum arabic (2–4% w/w) [63] but lower than that of *Khaya senegalensis* gum (5.25 ± 0.14%) [64]. This high value may not be detrimental to its use as tablet excipient since the acid-insoluble ash which constitutes soil contaminants (silica and silicates) that can cause abrasions on punches and dies of the tablet compressing machine is low.

Moisture content determination for potential pharmaceutical excipient(s) is a useful decisive formulation factor for formulation, storage, and shelf life of pharmaceuticals, especially water-sensitive active pharmaceutical ingredients (APIs) [51, 65]. Furthermore, with knowledge of moisture content, the formulation scientist is able to calculate the exact dry weight of the substance(s) to be used. As shown in Table 3, pCmG may be hygroscopic in nature and may be a good candidate for moisture-sensitive APIs, but when present in high concentration, products may have to be packed in a tight package material. The storage of this gum will not depend so much on temperature control [53].

As shown in Table 3, pCmG was soluble in distilled water at room temperature and 80°C and phosphate buffers of pH 7.4 and 6.8, representing pH of the small intestine and colon, respectively, and sparingly soluble in 0.1 N HCl in the stomach. The solubility of the gum was not affected by temperature. The lower solubility in 0.1 N HCl may be attributed to the weakly acidic nature of the gum or gum exhibiting pH response characteristics [66], and it is confirmed by the water absorptive parameters of the gum. All the values of absorptive parameters of the gum were lowest in the 0.1 N HCl medium. In the pharmaceutical formulation, the solubility profile of the potential excipient is very necessary as it suggests the type of dosage form appropriate for its use. From the results, appropriate manipulation of the concentration of pCmG can result in tablets that may resist dissolution in the upper GIT [67, 68].

The gum was practically insoluble in acetone, chloroform, and ethanol (96%). These are organic solvents in which many natural polysaccharides (gums) are insoluble [24, 69]. This insolubility behaviour also suggests that pCmG is an ionic gum [70].

Generally, gums exert their optimum functionality when sufficiently dissolved or hydrated in water [71]. All the water absorptive parameters (except distilled water) increased with increasing pH of the medium, suggesting disintegration and drug retention functional potentials of this gum when present in a solid dosage formulation. This gum will form highly viscous dispersions, and this is a characteristic of linear gums [9]. With all these absorptive properties of high HC, SI, and WRC in pH 7.4 (small intestine) than pH 6.8 (colon), pCmG may be a good drug delivery carrier for colon-targeting preparations and a good tablet disintegrant.

**Figure 1:**
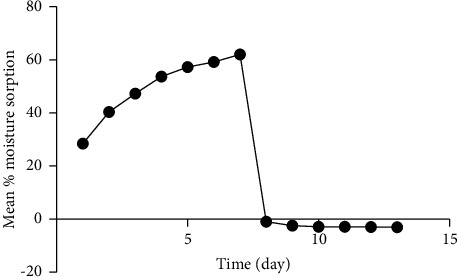
Moisture sorption capacity of pCmG.

The influence of extreme variations of storage conditions such as moisture on the solid-state stability of natural polymer powders was studied on pCmG using a moisture sorption-desorption pattern (Figure 1) [72]. At high humidity (100% RH) environment (room temp. 25°C), pGmG powder steadily adsorbed (sorption) significant quantities of moisture to reach the saturation point in 7 days. Even though sorption was rapid, it took 6-7 days to reach hydration equilibrium. This conforms to the general observation that hydration equilibrium time increases with %RH [73]. When the same moisture-saturated gum was transferred into an activated silica gel desiccator (extremely low humidity), there was a drastic loss of moisture (desorption). PCmG lost all sorption moisture (desorption) and a small portion of its moisture content within 24 h and continued to lose moisture to hydrous equilibrium on the 7^th^ day of pCmG in a desiccator containing the dried silica gel. As shown in Figure 1, water loss from pCmG was slow from day 8 to 13. This is because the hydrous equilibrium time is longer when water activity in the polymer is high [74]. Since the interaction of pharmaceutical excipients with water can affect some of their physicochemical and mechanical properties, this gum should not be exposed to very low humidity as loss of moisture may lead to excessive dryness that may affect these properties [75].

The total aerobic viable count or microbial load for pCmG was below BP [50] specification. All harmful organisms (*Staphylococcus* species, *Escherichia coli,* and *Salmonella typhii* species) [76] tested for were not detected. Moulds and yeast, usually considered as objectionable organisms, were detected but not in significant load as compared to BP [50] standards. On this basis, pCmG can be used as a pharmaceutical excipient in formulation processes and storage of the product, especially liquid dosage products [77].

Using the BP [50] scale of flowability, the angle of repose and Hausner ratio of pCmG powder were good and fair, respectively, with passable percent compressibility index. This means that if pCmG is included in tablet formulations, the granules may require glidants to obtain good granule flow during compression.

### 3.1. Tablet Formulation

#### 3.1.1. Gum-Drug (Paracetamol) Compatibility Testing

As shown in Table 4, the spectrum of freshly blended pCmG-paracetamol powders (I) revealed 3 main vibrational peaks of which one is the same as pCmG peak and the other two peaks are within the range of the functional groups in paracetamol. There was no indication of the formation of any new functional group, suggesting no chemical reaction and the resulting blend was just a mixture [78]. FTIR PAPs recorded from the spectrum of pCmG-paracetamol blend II, stored for 30 days at 40 ± 2°C at 75% relative humidity, depicted functional groups akin to the fresh blend (Table 4) and the percent concentration of paracetamol being the same. These are indications of the stability of the blend and no interaction thereof.

#### 3.1.2. pCmG Used as a Tablet Binder

Tables 5 and 6 summarize the micrometrics of granules and quality evaluation of compressed tablets of pCmG (Cm) as a binder. The gum was used in aqueous solutions of different concentrations as the granulating agent (Table 1).

As shown in Table 5, the percentage of fine granules was generally high in all the formulations, indicating low tackiness/binding properties of the gum. There was no trend as should have been expected.

Generally, the flow properties of granules of all the batches were poor; however, angles of repose were good (Table 5). These results agreed well with the flow behaviour of the gum powder; hence, the inclusion of magnesium stearate before compression was necessary.

Formulation batches Cm0.5 and Cm1.0 were too difficult to compress and the resulting tablets were too soft (Table 6) to merit further consideration. As shown in Table 6, only batches Cm2.0 and Cm4.0 with 2 and 4% w/w pCmG, respectively, passed the weight uniformity test. This inconsistency in the weight of these batches of tablets may partly be due to the flow properties of the granules. For resistance to crushing (RC) of the compressed tablets, only Cm3.0 and Cm5.0 were considered as passed. Tablets of all batches failed the friability test with high values. Tablets of all the batches passed the DT, even Cm5.0; the batch with the highest concentration (5% w/w) of gum passed the test. None of the batches passed all the quality evaluation tests. The binding properties of this gum were poor within the concentration range used and may be fully realized if the concentration is increased.

#### 3.1.3. Dissolution Profile of Compressed Tablets

**Figure 2:**
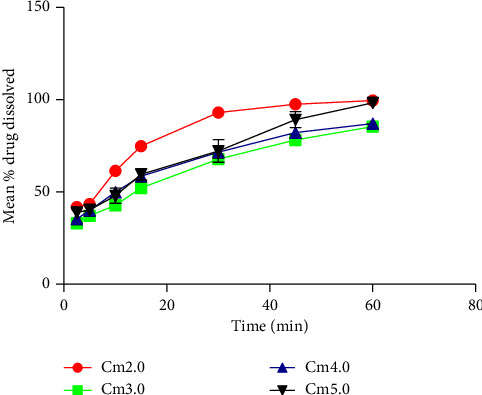
Mean % drug dissolved against time (min) for tablets with Cm as the binder.

Generally, there was a good drug (paracetamol) release within the tested period, as shown in Figure 2. All the dissolution curves in Figure 2 were evaluated with USP 30-NF 25 (2007), *T*_80_ standard, and model-independent approach, DE%, to obtain Table 7.

DE% values enable the effective distinction of drug release of the various batches [59, 79]. From DE%, drug release from the batches was in ascending order of Cm3.0, Cm4.0, Cm5.0, and Cm2.0 (Table 7). Tablets of batch Cm2.0 with the highest drug release had the fasted DT value of 5.2 min., friability of 15.31%, and RC value of 28.72 N which could not be effectively compared to Cm3.0, Cm4.0, and Cm5.0 which had relatively better hardness qualities (Table 6). Drug release from these 3 batches followed an increasing order of gum concentration, even though DT showed a decreasing order. Cm5.0 with the highest DT (14.37 min) and hardest 42.99 N among the batches of tablets had 98.58% drug release at 60 min dissolution. This may suggest pCmG to have some drug-releasing influence on these tablets, which therefore may be considered to be a potential good tablet binder and disintegrant at higher concentration.

With the USP 30-NF 25 (2007) standard, only tablets of batches Cm2.0 and C5.0 with the highest gum concentration and RC value passed the test (Table 7). This goes to confirm the earlier suggestion of the positive influence of pCmG on in vitro drug release of tablets.

#### 3.1.4. PCmG as Tablet Disintegrating Agent

Tables 8 and 9 represent concise descriptions of granules and quality assessments of tablets, respectively, when dry powders of pCmG (Cm) and standard corn starch (ST) were added to the tablet formulations as disintegrants. The flow characteristics of the granules of all the batches were almost uniform (Table 8). Using the BP [50] scale of flowability, batches with pCmG granules demonstrated fair flow for all parameters and corn starch granules showed varying flow parameters (Table 8).

All the batches of tablets containing gum and starch as disintegrants passed the RC and DT tests but failed the friability test. Tablets of all batches, except Cm8 and ST6, failed the uniformity of weight test at the lower limit.

**Figure 3:**
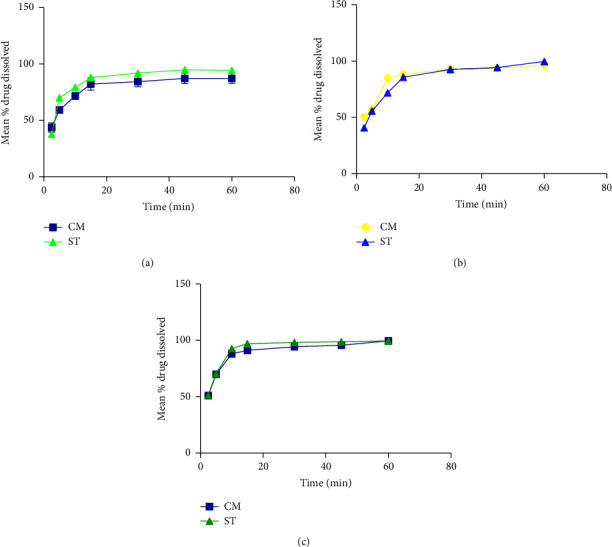
Mean % drug dissolved to time (min) for tablets containing (a) 6, (b) 8, and (c) 10% w/w disintegrating agent.

Drug release of ST6 tablets approached 100% and that of Cm6 was closer at the 60 min dissolution. Drug release patterns of ST6 and Cm6 were almost the same (Figure 3(a)). Increasing disintegrants concentration to 8% w/w demonstrated drug release patterns similar to the 6% w/w disintegrant tablets and higher release at each time point (Figure 3(b).

As shown in Figure 3(c), drug release of the 10% w/w disintegrant concentration tablets followed the same pattern as the 6 and 8% concentrations with highest drug release at each time point. There was almost 100% drug release for Cm10 and ST10.

#### 3.1.5. Dissolution Profile of Compressed Tablets Containing Cm and ST as Disintegrants

As shown in Table 10, DE% at 60 min for all the batches of tablets containing the same disintegrant was in the order of Cm6 < Cm8 < Cm10 and ST8 < ST6 < ST10. DE% values revealed the dependency of dissolution on tablet hardness for ST8 tablets. The dissolution rate of batches containing pCmG as disintegrant increased with concentration (Figure 3). Matching the DE values of batches, it was suggested that the disintegration action and drug release profile of tablets of pCmG and corn starch were comparable (Table 10).

*T*_80_ values of tablets of Cm batches decreased with increasing concentration of pCmG, indicating increasing disintegration of tablets into granules and/or deaggregation into primary drug particles for drug dissolution. Time for 80% drug dissolution was lower for the standard corn starch batches and followed a similar pattern as Cm batches (except ST8 batch).

Table 11 shows the concise quantification of the similarity of the compressed tablets of Cm and ST using the model independent mathematical difference factor (*f*_1_) or similarity factor (*f*_2_) [80].

The maximum *f*_1_ value for all tablets of Cm batches was 9.4%. This suggests that the mean maximum difference between drug release from tablets of Cm batches and the mean drug release of tablets of ST was about 9.4 at all the 7 time points of drug-released evaluations for batches Cm6 and ST6. This figure decreased further to 2.98 when the concentration of the disintegrant increased to 10% w/w (Table 11). This is an indication that the drug release profile of pCmG as disintegrant was not different from that of corn starch, especially at higher concentrations.

The similarity factor (*f*_2_) calculated confirmed drug release rates of Cm and ST to be similar and almost the same at higher concentrations (Table 11)

## 4. Conclusion

All the physicochemical properties concerning the formulation of conventional tablets studied about pCmG revealed good water absorptive parameters, suggesting disintegrating ability. No harmful organisms were detected in the gum, even though total aerobic viable count and objectionable organisms were detected within BP permissible limits, signifying that it can be used as a pharmaceutical excipient in oral formulations and will be generally preferred because of local availability and continuous supply. The tablet binding property of pCmG was poor but had a good disintegrating ability, similar to standard corn starch.

## Figures and Tables

**Table 1:** Formulae of pCmG-paracetamol tablets (100 tablets per batch).

Ingredients	Batch (g)					
	Cm0.5	Cm1.0	Cm2.0	Cm3.0	Cm4.0	Cm5.0
Paracetamol	50.00	50.00	50.00	50.00	50.00	50.00
Lactose	3.7	3.7	3.7	3.7	3.7	3.7
Starch	3.6	3.6	3.6	3.6	3.6	3.6
pCmG powder	0.29	0.58	1.17	1.77	2.39	3.02
Magnesium stearate (0.35%)^*∗*^	0.20	0.20	0.20^	0.21	0.21	0.21
Total weight	57.79	58.08	58.67^	59.28	59.90	60.53

^*∗*^Values were theoretical.

**Table 2:** Formula of 100 tablets of paracetamol with pCmG or starch powder as the disintegrant.

Ingredients	Formulations		
	A (6)	B (8)	C (10)
Paracetamol powder (g)	50.00	50.00	50.00
Lactose (g)	3.70	3.70	3.70
pCmG/corn starch powder (g)	3.43	4.67	5.97
PVP powder (2.5% w/w)	1.46	1.50	1.53

**Table 3:** Physicochemical, micromeritic, and microbial parameters of pCmG.

Parameters	Results
Organoleptic properties	
Colour	Light brown
Odour	Odourless
Taste	Bland/tasteless
Touch (powder sieve #80)	Smooth
Loss on drying (LOD)	7.33 ± 0.88
Solubility profile	
Distilled water (25°C)	Soluble
Hot distilled water (80°C)	Soluble
0.1 N HCl	Sparingly soluble
Phosphate buffer, pH 6.8	Soluble
Phosphate buffer, pH 7.4	Soluble
Acetone	Practically insoluble
Chloroform	Practically insoluble
Ethanol (96%)	Practically insoluble
pH (1% solution)	6.45 ± 0.06
Insoluble matter (%)	0.4989 ± 0.0024
Water absorption (%)	
Hydration capacity (HC)	
Distilled water	548.5 ± 4.36
HCl (0.1 N)	444.4 ± 10.17
Phosphate buffer, pH 6.8	588.8 ± 1.11
Phosphate buffer, pH 7.4	724.8 ± 12.76
Swelling index (SI)	
Distilled water	397.5 ± 7.54
HCl (0.1 N)	326.8 ± 14.06
Phosphate buffer, pH 6.8	497.0 ± 3.03
Phosphate buffer, pH 7.4	689.4 ± 30.4
Water retention capacity (WRC)	
Distilled water	55.67 ± 0.67
HCl (0.1 N)	49.33 ± 1.20
Phosphate buffer, pH 6.8	62.67 ± 1.86
Phosphate buffer, pH 7.4	77.00 ± 2.52
Inorganic constituents (ash values)	
Total ash	4.58 ± 0.24
Water-insoluble ash	2.05 ± 0.25
Acid-insoluble ash	1.07 ± 0.04
Ethanol-insoluble ash	3.05 ± 0.19
Micromeritics	
Fluff bulk density (*D*_*o*_) (g/ml)	0.607 ± 0.003
Consolidated density (*D*_*f*_) (g/ml)	0.790 ± 0.006
Hausner ratio	1.303 ± 0.003
Compressibility index (CI) (%)	22.87 ± 0.11
Angle of repose (°)	34.90 ± 0.157
Microbiological count (cfu/ml)	
Total aerobic viable	20
*Staphylococcus* species	Nil
*Escherichia coli*	Nil
*Pseudomonas* species	Nil
*Salmonella typhii* species	Nil
Yeast and mould (cfu/ml)	6

**Table 4:** Data of FTIR spectroscopy of (drug) paracetamol, CMPG, and the blended mixture.

PAP of drug (cm^−1^)	PAP of pCmG (cm^−1^)	SFG	Standard (cm^−1^)	PAP of pCmG-drugI (cm^−1^)	PAP of pCmG-drugII (cm^−1^)
3322.24	2992.80	Single bonds	3600–2700	2981.12	3321.81
3159.12		Aromatic C-H	3150–3070		3159.10
	2927.10	CH_2_	2930–2920		2920.86
	1699.42	Carboxylic acid	1725–1700		
		C=O			
1650.85		C=C	1655–1645		1650.94
1609.25		Aromatic ring	1610–1550	1609.25	1609.10
1561.35		C=N	1615–1580	1562.58	
	1462.12	C-O	1450–1350	1435.45	1435.06

PAP, principal absorption peak; SFG, suspected functional group; I and II, fresh and 30-day stored blends, respectively.

**Table 5:** Flow parameters of total paracetamol granules prepared with pCmG as the binder.

Batch	% fines	Hausner ratio	Compressibility index (%)	Angle of repose (°)
Cm0.5	29.3204	1.2033	16.8976	33.60 ± 0.32
Cm1.0	27.1204	1.2998	23.0694	36.55 ± 0.62
Cm2.0	25.5604	1.2547	20.3010	39.34 ± 0.39
Cm3.0	23.6139	1.1922	16.1206	36.03 ± 0.64
Cm4.0	26.2602	1.2308	18.7489	33.95 ± 0.51
Cm5.0	25.6100	1.2916	22.5760	36.75 ± 0.77

**Table 6:** Quality of the compressed tablets containing varying quantities of pCmG in solution.

Batch	Mean weight (g)	Weight deviation			RC (N)	Friability (%)	DT (min.)^*∗*^	Drug content (%)
		Tablet(s) deviated		SD	±SD^*∗*^			
		>5%	>10%					
Cm0.5	0.5325	3	2	0.0322	23.42 ± 8.35	16.53	1.68	101.55
Cm1.0	0.5622	3	2	0.0222	24.76 ± 8.58	20.19	2.72	101.54
Cm2.0	0.5748	1	0	0.0282	28.73 ± 4.55	15.31	5.2	101.98
Cm3.0	0.5319	3	1	0.0486	41.58 ± 15.63	5.14	5.55	103.10
Cm4.0	0.5584	0	0	0.0226	38.85 ± 5.34	9.68	6.72	100.63
Cm5.0	0.5893	4	0	0.0374	42.99 ± 8.18	9.31	14.37	101.23

^*∗*^ *N* = 6; RC = resistance to crushing; DT = disintegration time.

**Table 7:** Mean dissolution parameters of compressed tablets.

Formulation batch	Parameters	
	Mean T_(80)_ (min)	Mean DE (%)
Cm2.0	20.40 ± 0.04	84.82 ± 0.07
Cm3.0	46.86 ± 0.11	65.17 ± 0.04
Cm4.0	39.25 ± 0.17	69.38 ± 0.06
Cm5.0	24.81 ± 0.10	80.62 ± 0.06

**Table 8:** Flow parameters of total granules of pCmG and starch as disintegrants.

Batch (%)	% fines	HR	CI%	Angle of repose (°)
Cm5	28.075	1.200	16.658	37.40 ± 0.58
Cm8	24.410	1.201	16.712	37.20 ± 0.70
Cm10	24.391	1.192	16.177	37.29 ± 0.36
ST5	21.325	1.207	17.137	37.47 ± 0.19
ST8	21.384	1.228	18.578	33.06 ± 0.12
ST10	24.401	1.228	18.566	35.48 ± 0.50

**Table 9:** Quality of the compressed tablets containing gums as the disintegrating agent.

Batch	Mean tablet weight (g)	Weight deviation			RC (Newton)	Friability (%)	DT (min)
		Tablet(s) deviated		SD	± SD^*∗*^		
		>5%	>10%				
Cm6	0.6240	6	1	0.0562	70.50 ± 8.31	1.94	5.33
Cm8	0.6240	1	0	0.0275	78.33 ± 11.39	1.68	8.20
Cm10	0.6212	4	0	0.0400	67.11 ± 14.48	1.20	5.63
ST6	0.6122	2	0	0.0322	67.81 ± 17.02	1.36	4.95
ST8	0.6609	4	0	0.0359	90.53 ± 10.58	3.86	3.78
ST10	0.6567	4	0	0.0367	67.37 ± 14.66	1.10	5.40

^*∗*^ *N* = 6.

**Table 10:** Mean dissolution parameters of tablets.

Formulation batch	Parameters	
	Mean T_(80)_ (min)	Mean DE (%)
Cm6	14.92 ± 1.20	81.45 ± 4.06
ST6	14.94 ± 0.23	88.56 ± 0.45
Cm8	14.48 ± 0.0	88.86 ± 0.07
ST8	17.41 ± 0.07	87.42 ± 0.04
Cm10	11.95 ± 0.0	91.77± 0.0
ST10	9.9 ± 0.0	95.05 ± 0.06

**Table 11:** Data of difference (*f*1) and similarity factors (*f*2) of batches of tablets with pCmG and corn starch as disintegrating agents.

Formulation batch	Difference factor (*f*1)	Similarity factor (*f*2)	Comment
Cm10	2.98	97.25	Similar
Cm8	5.67	59.39	Similar
Cm6	9.40	55.71	Similar

## Data Availability

The data used to support the findings of this study are included within the article.
